# Supplementary material for: Computational Eurotransplant kidney allocation simulations demonstrate the feasibility and benefit of T-cell epitope matching
Source: PLoS Comput Biol. 2021 Jul 27;17(7):e1009248. doi: 10.1371/journal.pcbi.1009248 (PMC8345832; doi:10.1371/journal.pcbi.1009248)
Supplement: S1 Text — (DOCX) [file pcbi.1009248.s001.docx]

# Computational Eurotransplant kidney allocation simulations demonstrate the feasibility and benefit of T-cell epitope matching – supporting information

Matthias Niemann* (1), Nils Lachmann (2), Kirsten Geneugelijk (3), and Eric Spierings (3)

(1) PIRCHE AG, Berlin, Germany

(2) Center for Tumor Medicine, H&I Laboratory, Charité University Medicine Berlin, Berlin, Germany

(3) Center of Translational Immunology, UMC Utrecht, Utrecht, The Netherlands

* [matthias.niemann@pirche.com](mailto:matthias.niemann@pirche.com)

## S1 Text: Evaluation of ETKASPIR-A - ETKASPIR-D

In the following, we describe the stepwise improvement of the ETKASPIR models over the ETKAS simulation.

### Prioritization of low PIRCHE-II scores (ETKASPIR-A)

The first modification to the ETKAS simulation model was replacing the full HLA-match prioritization by a PIRCHE-II based prioritization. S1B Fig shows the PIRCHE-II score density plot of the resulting ETKASPIR-A model. Implementation of PIRCHE-II based prioritization indeed increases allocations in the lowest PIRCHE-II range and reduces the number of allocations in the second PIRCHE-II interval. The depicted shift is reflected by the changes in the respective areas under the curve (AUC) and distribution-weighted mean log(PIRCHE-II) values (WML) (S1 Table). This shift is accompanied by a reduced number of HLA-A, -B, -DR matched combinations, with 22.1% matched situations in the ETKAS allocation simulations and 12.1% matched situations in the ETKASPIR-A simulations.

**
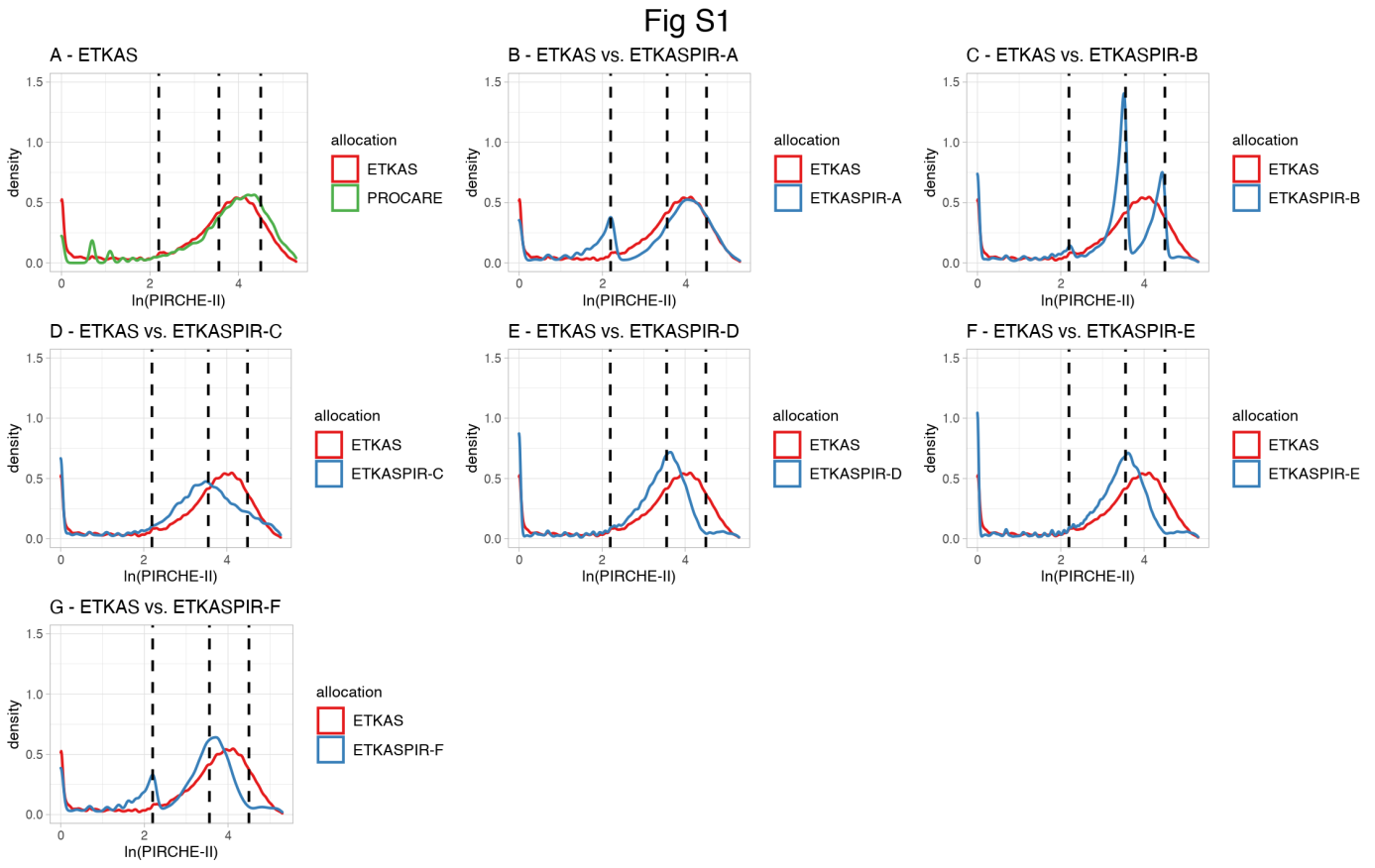
**

**S1 Fig: The log-transformed PIRCHE-II score distributions resulting from the different ETKAS simulation models.**

*Density plots for the simulations were generated using a Gaussian smoothing kernel. Red lines in graphs A-G represent the PIRCHE-II scores in the ETKAS baseline simulations, the green line in graph A represents the previously reported PIRCHE-II scores as observed in the PROCARE cohort* [1]*, blue lines in graphs B-G represent the PIRCHE-II scores in the PIRCHE-II modified ETKAS simulations for the ETKASPIR-A model with priority allocation by PIRCHE-II score (B), ETKASPIR-B with priority allocation by HLA match and points distributed according to previously reported PIRCHE-II ranges (C), ETKASPIR-C with priority by HLA match and points distributed exponentially descending (D), ETKASPIR-D with priority by HLA match and assignment of points linearly descending (E), ETKASPIR-E with priority by HLA match, assignment of points linearly descending, and inclusion of a PIRCHE-II based mismatch probability (MMP) (F), and ETKASPIR-F with priority allocation by PIRCHE-II score, assignment of points linearly descending, and inclusion of a PIRCHE-based MMP (G). Dashed lines indicate the previously reported strata by Lachmann et al* [2]*.*

**S1 Table: PIRCHE-II distribution per risk strata in percent.**

|  |  | PIRCHE-II score ranges | | | |  |
| --- | --- | --- | --- | --- | --- | --- |
|  |  | Group 1  0-9  (WML) | Group 2  9-35  (WML) | Group 3  35-90  (WML) | Group 4  > 90  (WML) | p value  (SWML) |
| Allocation model | PROCARE | 10.87 %  (0.095) | 21.62 %  (0.672) | 47.71 %  (1.937) | 19.81 %  (0.953) | < 0.01^I^  (3.657) |
|  | ETKAS | 15.52 %  (0.112) | 25.08 %  (0.784) | 46.30 %  (1.871) | 13.10 %  (0.624) | -  (3.391) |
|  | ETKASPIR-A | 29.63 %  (0.406) | 13.12 %  (0.425) | 43.51 %  (1.765) | 13.73 %  (0.655) | < 0.01^I^  (3.250) |
|  | ETKASPIR-B | 19.78 %  (0.162) | 42.90 %  (1.425) | 32.98 %  (1.401) | 4.33 %  (0.214) | < 0.01^II^  (3.202) |
|  | ETKASPIR-C | 18.81 %  (0.141) | 38.70 %  (1.190) | 30.79 %  (1.225) | 11.70 %  (0.570) | < 0.01^III^  (3.127) |
|  | ETKASPIR-D | 17.38 %  (0.119) | 40.93 %  (1.284) | 37.49 %  (1.455) | 4.20 %  (0.209) | < 0.01^IV^  (3.067) |
|  | ETKASPIR-E | 17.17 %  (0.116) | 41.66 %  (1.308) | 36.13 %  (1.404) | 5.04 %  (0.256) | 0.83^V^  (3.083) |
|  | ETKASPIR-F | 27.12 %  (0.353) | 30.93 %  (0.993) | 37.02 %  (1.444) | 4.92 %  (0.244) | 0.02^V^  (3.034) |

*Pairwise comparisons of PIRCHE-II scores using Wilcoxon’s rank sum test considered (I) ETKAS, (II) ETKASPIR-A, (III) ETKASPIR-B, (IV) ETKASPIR-C and (V) ETKASPIR-D. Distribution-weighted mean log(PIRCHE-II) per PIRCHE-II range (WML) in parentheses. Sum of distribution-weighted mean log(PIRCHE-II) values (SWML) integrate frequency and PIRCHE-II distribution.*

### Stratified PIRCHE-II matching score (ETKASPIR-B)

Next, we replaced the HLA matching score in the ETKAS simulation model with a PIRCHE-II based score using the categories that we previously defined [2]. To this end, a maximum of 400 points was assigned to the four discrete PIRCHE-II groups as described above and as depicted in Fig 3. The resulting ETKASPIR-B model resulted in an increased number of allocations in group 1 and group 2, in favour of the high-risk groups 3 and 4 (S1C Fig, and S1 Table). The ETKASPIR-B model results in an overall effect similar to the ETKASPIR-A model, as reflected by similar sum of distribution-weighted mean log(PIRCHE-II) (SWML) scores (3.250 for ETKASPIR-A and 3.202 for ETKASPIR-B). Yet, the values of the WML scores were different. Allocation into group 1 is lower in the ETKASPIR-B model when compared to ETKASPIR-A (19.78% and 29.63% respectively). However, the ETKASPIR-B model strongly enhances allocation into group 2 (42.90%; WML=1.425; S1 Table).

Although the improvements were highly significant in a stratified way when compared to the ETKAS simulation model, we observed an undesirable accumulation of allocations at the upper limits of the quartiles, reflected by peaks in the density plots near the PIRCHE-II cut-off values of 9, 34, and 90 respectively. These accumulations indicate that, within each of these individual three groups, there is a skewing to the higher risk combinations.

### Continuous negative exponential PIRCHE-II matching score (ETKASPIR-C)

A continuous scoring system will eliminate the accumulation of allocations just below the set limits, as observed for the ETKASSPIR-B model. We therefore applied a negative exponential function to weigh the PIRCHE score in a continuous way. Indeed, the accumulation effects were resolved by using this model (S1D Fig). However, relative to the ETKASPIR-B model, we observed relatively more allocations in the high-risk group 4 for the ETKASPIR-C model (S1 Table; 4.33%; WML=0.214 versus 11.70%; WML=0.570). Thus, the ETKASPIR-C model is less effective in avoiding high-risk allocations.

### Continuous inverse linear PIRCHE-II matching score (ETKASPIR-D)

In the ETKASPIR-D model, PIRCHE-II based scores assignment of allocation points in the range of 0-90 was executed linearly descending. Allocations with a PIRCHE-II score above 90 were awarded zero points, with the intention to avoid recipient-donor combinations ending up in the high-risk group 4. This model significantly reduces the percentage of allocations in group 3 and 4 when compared to the baseline ETKAS model (S1E Fig). In parallel, the WML scores strongly improved. The overall positive effect of this model is reflected by a highly reduced SWML when compared to the ETKAS simulation model (S1 Table). Based upon the WML scores and the SWML scores, we concluded that the ETKASPIR-D model is the most suitable variant for assigning a PIRCHE-II based allocation score.

### Introduction of the PIRCHE-II-based mismatch probability (ETKASPIR-E)

The HLA mismatch probability is a scoring tweak that compensates individuals with HLA typings that are difficult to match and intends to result in acceptable waiting times for these individuals. The incompatibility between the PIRCHE-II based matching score and the serological typing based HLA mismatch probability (MMP) that ET is currently applying, may corrupt the correct balance between these two scoring aspects. We therefore evaluated whether this was the case and whether the inclusion of the PIRCHE-II Risk Profile (PIRCHE-II RP) would be able to correct that.

S2 Fig displays the mean waiting times in the ETKASPIR-D model. After 10 simulated years in this model, virtual recipients on simulated waiting lists with a PIRCHE-II RP median of more than 200 appear to have a significantly extended waiting time of 308 days (mean waiting time = 2152 days, SD = 1138 days) when compared to those with a PIRCHE-II RP median of equal to or below 200 (mean waiting time = 1844 days, SD = 1103 days, p < 0.001). Thus, although the ETKASPIR-D model optimizes for low risk transplants, it seems to significantly harm a fair waiting time for all individuals on the waiting list.

***
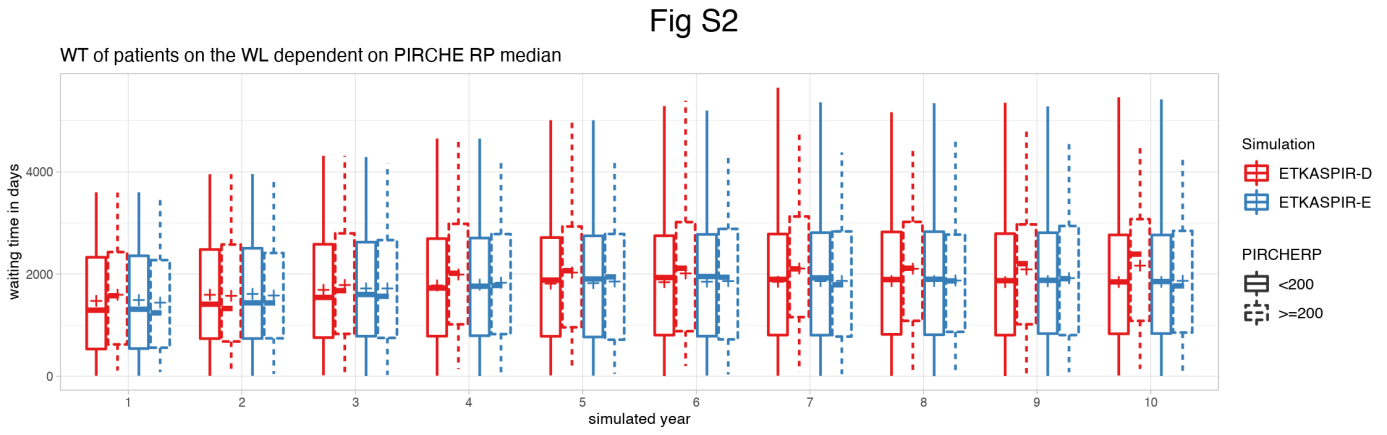
***

***S2 Fig: Waiting time distributions of patients***

*Waiting time distributions of patients in ETKASPIR-D (red) and ETKASPIR-E (blue) during the simulation. Solid boxes describe the subset of patients with a PIRCHE-II Risk Profile score <= 200, dashed boxes describe the subset of patients with a PIRCHE-II Risk Profile score > 200. Respective mean waiting times are shown as circles. To facilitate readability, a dodge function was applied to the x-axis. Boxplot depicts the median (horizontal line), mean (plus) and first to third quartile (box), the highest and lowest value within 1.5x IQR (whiskers).*

Given the negative effects on waiting time for individuals with a high PIRCHE-II RP median in the ETKASPIR-D model, we considered the PIRCHE-II RP median in the ETKASPIR-E simulations by including the PIRCHE-II MMP. The inclusion of the PIRCHE-II MMP had no significant effect on the overall allocation, with similar proportions in the 4 risk groups and similar WML and SWML scores (SWML=3.083; p=0.83; S1 Table). Importantly, as shown in S2 Fig, the implementation of a PIRCHE-II MMP led to equal waiting times for both the individuals with a high and for those with a low PIRCHE-II RP medians. (<= 200: mean waiting time = 1845 days, SD = 1203 days; > 200: mean waiting time = 1867 days, SD = 1127 days; p = 0.66). Thus, inclusion of a PIRCHE-II based MMP score rather than a classical HLA matching-based MMP seems to be essential in order to create fair chances for all recipients on the waiting list.

### Blood group distributions

ETKAS considers blood group identical donors for patients, causing the blood group distribution of transplanted recipients being identical to the donor blood group distribution (S3 Fig). However, as the distributions suggest fewer blood group 0 donors compared to recipients, there are proportionally more waiting patients with blood group 0.

**
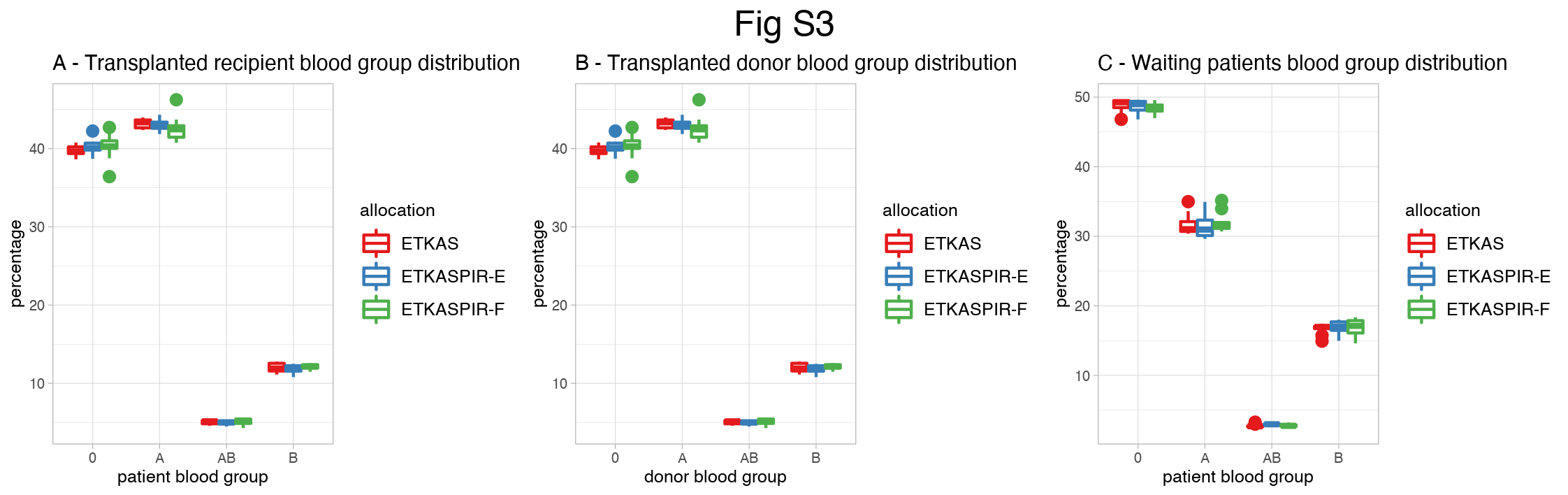
**

**S3 Fig: Blood group distributions**

*Blood group distributions within ETKAS (red), ETKASPIR-E (blue) and ETKASPIR-F (green) for transplanted patients (A), transplanted donors (B) and remaining patients on the waiting list (C).*

## References

1. Geneugelijk K, Niemann M, Drylewicz J, van Zuilen AD, Joosten I, Allebes WA, et al. PIRCHE-II Is Related to Graft Failure after Kidney Transplantation. Front Immunol. 2018;9:321.

2. Lachmann N, Niemann M, Reinke P, Budde K, Schmidt D, Halleck F, et al. Donor-Recipient Matching Based on Predicted Indirectly Recognizable HLA Epitopes Independently Predicts the Incidence of De Novo Donor-Specific HLA Antibodies Following Renal Transplantation. Am J Transplant Off J Am Soc Transplant Am Soc Transpl Surg. 2017 Dec;17(12):3076–86.
