# Supplementary material for: Computational Eurotransplant kidney allocation simulations demonstrate the feasibility and benefit of T-cell epitope matching
Source: PLoS Comput Biol. 2021 Jul 27;17(7):e1009248. doi: 10.1371/journal.pcbi.1009248 (PMC8345832; doi:10.1371/journal.pcbi.1009248)
Supplement: Attachment — Submitted filename: Point by point reply.docx [file pcbi.1009248.s002.docx]

Dear Editors, dear Reviewers,

We appreciate the review of our manuscript. Based on your helpful comments and suggestions, we improved the manuscript and provide further data. The corresponding changes in the manuscript are highlighted with a yellow background. Thank you very much for considering our manuscript further.

Sincerely

Matthias Niemann

# Point-by-point reply:

### Reviewer #1:

### "First, a virtual waiting list was constructed by bootstrapping, using the Eurotransplant Annual Report 2015."

### You need to provide a great deal more information on how you performed your simulation, since all of your conclusions hinge on it. Since the Eurotransplant Annual Report provided no individual patient data, how did you 'bootstrap' a waitlist?

We thank Reviewer #1 for stressing this point and agree. In concordance with Reviewer #2, we swapped the Methods and Materials section with the Results section, so the simulation and its bootstrapping are explained before diving into the result. Also, we moved the reference to the detailed protocol ([dx.doi.org/10.17504/protocols.io.bqrtmv6n](https://dx.doi.org/10.17504/protocols.io.bqrtmv6n)) to lines 246f:

*“A detailed protocol of the initialization, the bootstrapping and the simulation steps is available online at https://dx.doi.org/10.17504/protocols.io.bqrtmv6n”*

To support the reader in understanding the applied steps, a schematic was added as fig 1, lines 187ff.

**Fig 1**: **Simulation schematic**

*During the bootstrapping process, virtual patients and donors were created based on haplotype frequencies. The initial waiting list was randomly populated matching the distributions provided by Eurotransplant. During the simulation, waiting patients were randomly removed from the waiting list (e.g. unfit for transplantation, died waiting) and new patients were randomly registered to the waiting list. Virtual donors were randomly registered and allocated to the current waiting list resulting in simulated transplantations.*

Indeed, ET reports do not provide individual patient data. Therefore we decided to apply the simulations with virtual individuals for this project. A follow-up project in collaboration with Eurotransplant actually repeats these simulations with historical patient and donor data. Preliminary results suggest similar findings:

Lachmann, N., Niemann, M., Tieken, I., Halleck, F., Geneugelijk, K., Spierings, E., & Budde, K. (2020, October). IN SILICO SIMULATIONS DEMONSTRATE THE BENEFIT OF HLA EPITOPE MATCHING FOR KIDNEY ALLOCATION. In *TRANSPLANT INTERNATIONAL* (Vol. 33, pp. 32-33). 111 RIVER ST, HOBOKEN 07030-5774, NJ USA: WILEY.

<https://doi.org/10.1111/tri.13733>, PO-95

### Bootstrapping usually involves constructing random samples with replacement of a set of individual observations. How did you manufacture counterfactual patient histories? For example, suppose a patient had originally received a transplant in March, but your simulation did not give them a transplant until December (if at all). How do you know the patient is still alive? If you did not construct these histories, then it is a foregone conclusion that your approach would appear (in your simulations) to have better outcomes; patients who never received a transplant under your allocation system would be immortal. Note: please do not use Markov models to simulate patient histories, as patient medical histories completely violate the 'memoryless' assumption that Markov models depend on.

We agree with Reviewer #1, that real-world patient histories may cause anomalies in the simulation due to differing decisions of the simulation of the real-world. However, in our simulation with virtual individuals, patient histories are defined on demand, so there is no mismatch to a predefined or expected event. While waiting for a transplantation, all patients on the virtual waiting list have a chance to be removed from the waiting list without being transplanted (e.g. unfit for transplantation, personal decision or died waiting) based on the ET-reported frequency of such event (lines 239ff).

With the changes above, we took care of this in the Materials and Methods. Furthermore, we added in the Discussion, lines 714f:

*“We therefore suggest that such data could only be included based upon real-life allocation data as stored by the various allocation organisations, considering the difficulties to apply Markov models to real patient histories.”*

### "Subsequently, for the non-HLA typing characteristics, the parameters country, urgency, recipient age, blood group, percentage of PRA, and waiting time were extracted from the Eurotransplant Annual Report 2015 39 and assigned to the virtual waiting list population accordingly. Country, urgency and age were considered as independent parameters and thus assigned randomly, using the reported overall frequencies. Distributions for blood group, PRA, and waiting time were considered conditional to the virtual individuals’ country and thus assigned according to country-dependent frequencies. "

### This contradicts the above statement, and implies that you assigned random ages, blood groups, PRA percentages, and waiting times. How did you handle the inter-dependent factors? Women are more likely to be older, and they're more likely to be highly sensitized. Older patients are less likely to survive long periods of time on the waitlist. How does blood group relate to HLA? Generally speaking, it is not a good idea to consider two demographic or medical variables to be completely independent of each other.

Indeed, random values were assigned based on distributions provided by ET reports. Unfortunately, not all of the provided data was dependent on relevant confounders (e.g. on age and gender). ETKAS and our simulated version of it does not consider gender and we do not observe gender as an outcome parameter. Moreover, the increased likelihood of women for pre-sensitization is reflected by PRA distribution within the cohort. Consequently, for gender this issue does not apply here. However, the reviewer is correct that the age-dependent probability of survival on the waiting list is not considered and hampers the prediction. Conversely, ignoring the correlation between certain HLA types and blood groups may cause the simulation to underestimate HLA match grades compared to reality. We added this correlation to the list of incomplete factors in the discussion (lines 703ff):

*“Other factors that were absent or incomplete in our simulations are the increasing genetic diversity in patients and donors over time for instance due to migration, temporal aspects of immunization, specific center policies, rejected graft offers due to positive crossmatches, organ quality, accuracy of demographic parameters, deregistrations dependent on specific factors, dependency of HLA type and blood group, and the spatial resolution as now defined on country level rather than region.”*

We accepted this as a weakness of the study design. Specifically for that reason, we do not compare our simulated results with the Eurotransplant-reported outcomes but execute a baseline simulation to which our modified allocations were evaluated against. The systematic error of that approach applies both to the baseline simulation and modified iterations, so its impact remains constant. We added this clarification in the discussion (lines 709ff):

*“These inaccuracies limit comparability between simulation and real data. However, as we applied a baseline simulation to compare our modified allocation models, the error is systematic and applies similarly to all simulated instances. Such [...]”*

### "our simulations of the Eurotransplant Kidney Allocation System suggest an increasing absolute incidence of a functioning graft 10 years after transplantation by 1.1% to 1.5%. This absolute increase translates into a relative improvement of 12.9% to 13.5% bearing in mind the spread in graft survival at 10 years between low and high scores being 8% to 12%"

### You do not describe how you estimated this survival improvement, although you imply it's based solely on matching. Kidney and patient survival is affected by a great deal more than just antigen and epitope matching. Donor and recipient demographics (especially age) comorbidities and other case mix factors all have substantial impact on patient and graft survival. Any statement that a new allocation system would improve survival needs to do more than just look at matching. Does epitope matching affect racial allocation? If so, then changing the allocation may be affecting disparities in racial access to transplantation, and thus survival. Similarly, if patients had to wait longer for kidneys, then the increased dialysis time is likely to reduce post-transplant survival.

We agree with the reviewer, that our estimations of graft survival improvement is not considering other factors involved. Most of these factors are however not part of the simulation and therefore by design do not have an effect on the simulation. Vice-versa, the considered literature (Lachmann et al. and Geneugelijk et al) did not provide data fine-grained enough (except for donor and recipient age) to comprehensively consider such factors as underlying confounders. Donor age was not considered by the simulation and is not considered by ETKAS. However, in reality there could be a bias clinically and also from allocation perspective, as kidneys from old donors may be more frequently accepted by surgeons for an old patient opposedly to a pediatric patient. We explained this estimation in the Materials & Methods (lines 400ff):

*“Estimation of improved graft survival considered reported univariate incidences of graft survival at 10 years post transplant in the respective PIRCHE-II groups* [*^18,19^*](https://www.zotero.org/google-docs/?Wv3Dkx)*. PIRCHE-II group frequencies of simulations were multiplied with respective graft survival and summed across all groups.”*

Furthermore, we added to the discussion (lines 625f):

*“However, confounding factors outside the scope of our simulations may affect these estimates, necessitating further modeling studies.”*

In our simulation results we didn’t observe significant changes in the distributions for recipient age (Fig 4F) or waiting time (both on the waiting list (7C) and the transplanted patients (7B)). Therefore we wouldn’t expect an impact of these parameters on graft survival. We added a plot of the recipient age as a panel to figure 4 and referenced it in the Results section (lines 462ff):

*“In the different allocation simulations, there was no impact on the transplanted recipients’ age observed considering the individual PIRCHE-II score groups (Fig 5F).”*

We think it is not to be expected that epitope matching is more prone to “racial allocation” than plain serologic matching. With mismatch probability points, ET already incorporated a concept to avoid that effect. The epitope-equivalent as suggested in our manuscript (PIRCHE risk profile median score) should yield the same impact. However, we agree this issue has to be addressed appropriately by simulations or observations. Accordingly, we added that limitation to the discussion (lines 700ff):

*“Although we suggest the epitope-equivalent PIRCHE-II RP mechanism over the MMP to promote patients with rare genotypes, further studies are warranted to investigate a potential disadvantage for certain patient groups or populations introduced by epitope matching*, e.g. by considering real-world donor and recipient typing data*.”*

The increased kidney exchange however may potentially yield in longer cold ischemic time considering longer distances to be travelled. This would indeed have a negative impact on graft survival. We added that fact to the discussion (lines 746f):

*“An increasing exchange might indicate longer average cold-ischemic times, which impairs outcome.”*

### "We first performed an allocation in triplicate using the unmodified ETKAS procedure as described in the Eurotransplant ETKAS Manual fig 7." Your statement that you used an unmodified ETKAS allocation system is incorrect. You omitted the ETKAS allocation rules for highly-sensitized candidates. Why? This is an important group that would likely be strongly impacted by changes in matching allocation rules.

Eurotransplant distinguishes its allocation programs between Eurotransplant Kidney Allocation System (ETKAS), Acceptable Mismatch program (AM) and Eurotransplant Senior Program (ESP). These programs are considered individually also in ETs annual reports. Plain ETKAS, therefore does not include the allocation of highly-sensitized patients allocated via AM. This was specifically done to show the impact of epitope matching on the largest allocation program compared to the very low throughput allocation of AM. We added a clarification to the introduction (lines 129f):

*“ETKAS is the largest kidney allocation program of Eurotransplant with around 70% of the donors being allocated by it* [*^11^*](https://www.zotero.org/google-docs/?GEVwOs)*.”*

Furthermore, we clarified in the Materials & Methods (lines 270f):

*“We first performed an allocation in triplicate using the unmodified ETKAS procedure as described in the Eurotransplant ETKAS Manual fig 2* [*^26^*](https://www.zotero.org/google-docs/?hMrpga)*, assuming an HLA genotype-independent outflow of donors to the AM program.“*

We agree with the reviewer, the AM recipients are a very interesting cohort, having very special patient histories. Consequently, we decided to exclude AM from simulations as modeling patients requires large amounts of data - or preferrably access to real patients’ antibody history. We clarified this in the discussion (lines 717ff):

*“With sufficient real-life data of complex, highly-sensitized recipients, simulations could also be extended to estimate the impact of the AM program, which intentionally was excluded in our simulation with virtual individuals.”*

### Would your allocation system affect the blood-type distribution of recipients? O-type recipients are already disadvantaged; would your system exacerbate this?

We share the reviewer’s concern about disadvantages of recipients with certain blood group. Eurotransplant adjusted ETKAS from a blood group compatible paradigm to a blood group identical concept in 2010, to avoid unfair advantages of certain recipients. Our modifications were solely based on histocompatibility factors, leaving the blood group mechanism unchanged (fig 2). With required blood group identity, the blood group distribution of transplanted recipients is only dependent on donor blood group. We added figure S3 accordingly to the supplement, showing the blood groups in the different simulated allocations. Given the lower proportion of blood group 0 donors compared to the proportion of blood group 0 patients on the waiting list, these patients were disadvantaged in all simulated allocations.

### Did your allocation system use all the kidneys, or were there changes in whether some of the marginal kidneys were used or discarded?

The concept of discarding kidneys due to organ-specific reasons was not considered in our simulations, as it has no effect on the demographics of transplanted patients. Consequently, all donor organs that were registered were eventually transplanted. The simulations were configured with the number of transplanted donor organs as provided by the ET report - not with the number of registered donors.

Lacking reliable data on recipient-specific reasons to turn down an organ offer, declining was modeled independently of other factors (age, match grade, center policy, etc.) with a constant 10% chance.

In the discussion, we added organ quality to the list of absent concepts of our simulations (lines 703ff):

*“Other factors that were absent or incomplete in our simulations are the increasing genetic diversity in patients and donors over time for instance due to migration, temporal aspects of immunization, specific center policies, rejected graft offers due to positive crossmatches, organ quality, accuracy of demographic parameters, deregistrations dependent on specific factors, dependency of HLA type and blood group, and the spatial resolution as now defined on country level rather than region.”*

### Your simulation references are fairly incomplete. There has been a fair amount of work done in the past twenty years to develop adequate organ allocation simulations. Please review more of the literature. Some of the American work done by the SRTR might be helpful.

We thank the reviewer for pointing this out. We adjusted the manuscript to specifically refer to the SRTR and added references to further literature based on the SRTR data and simulation software.

lines 634ff:

*“The KPSAM software considers patient and donor data from the Scientific Registry of Transplant Recipients (SRTR) and was instrumental in the 2014 introduction of the kidney donor profile index (KDPI) by the Organ Procurement and Transplantation Network (OPTN) into a new national deceased donor kidney allocation* [*^39^*](https://www.zotero.org/google-docs/?WHaHWx)*.”*

lines 641ff:

*“After the implementation and validation of the current OPTN kidney allocation, KPSAM is still beneficial to evaluate further options to improve utility and equity of kidney allocation e.g. for highly-sensitized recipients* [*^40^*](https://www.zotero.org/google-docs/?OrijXR)*. Consequently, SRTR data and simulated allocation models are also applied in lung and liver transplantation, with each setting having its unique allocation system requirements* [*^41,42^*](https://www.zotero.org/google-docs/?b2AllJ)*. “*

### Reviewer #2: Thank you for your very interesting and promising paper. In its current presentation, the result section is quite difficult to read and to understand. Reading the results section before the methods section make the results and their relevance very difficult to understand indeed. This make you paper falsely looking more a technical report than a scientific one.

We thank the reviewer for the comments and agree with the concerns about the structure. As suggested by point 4, we put the Methods section before the Result section to improve readability.

### If one consider the title, your are supposed to demonstrate the feasibility and the benefit of integrating the T-Cell epitope matching within ETKAS.

### 1) For this purpose, do you think it is really necessary to have results for 8 allocations models including 6 simulated ones ? What does PROCARE add to the demonstration ? Of course ETKAS is required but I suggest you to select the minimum of simulations required to the demonstration. You could explain you had a stepwise approach whose results are provided as supplementary material.

We thank the reviewer for the suggestion and extracted the results of ETKASPIR-A, -B, -C, and -D to a supplement, so the reader may concentrate on the final models. We decided to keep the PROCARE cohort in the manuscript as a validation cohort for the ETKAS simulation. It shows the reader that our baseline simulation matches a real-world cohort of transplantations being carried out in the ET region.

Accordingly, we removed panels of the provided figures and parts of the provided tables in favor of adding this data to the supplementary material.

We reformulated the result section for ETKASPIR-E accordingly (lines 491ff):

The modified allocation approaches ETKASPIR-A to -D were evaluated with their individual components’ impact on allocation as demonstrated in the supplementary material. Based on these findings, ETKASPIR-E was constructed. Compared to the baseline model, ETKASPIR-E increases the number of transplantations being carried out with lower PIRCHE-II scores (fig 6B). The number of transplantations with very low PIRCHE-II scores is only slightly increased over ETKAS (17.17 % vs. 15.52 %; table 2). However, due to more transplantations being carried out with intermediate-low PIRCHE-II scores (41.66 % vs. 25.08%, table 2) and a therefore decreased number of transplantations with intermediate-high and high scores, the SWML reduces significantly to 3.083 compared to 3.391 of ETKAS (p < 0.001).

### 2) Gaining place, you could then explain more the evaluation metrics you are using, especially in terms of the feasibility and benefit you are supposed to demonstrate. What are metrics to challenge the realism of the simulation (data generation, process, allocation scheme). What are the metrics used to validate the feasibility and those for the benefit.

We kindly thank the reviewer for the suggestion and added a section to the Materials and Methods describing the applied metrics (lines 372ff):

### **Evaluation metrics**

*Validity of the bootstrapping process was evaluated by observing the distributions of patients’ blood group, PRA, waiting time, age and country. To evaluate the realism of the baseline simulations, PIRCHE-II distributions were compared to an actual transplant cohort. Additionally, HLA match grade distributions were compared to the baseline simulation.*

*The benefit of the modified allocation of the status quo simulation was evaluated by calculating the respective areas under the curve (AUC) in previously reported PIRCHE-II ranges knowingly correlated to outcome. PIRCHE-II distributions of simulated transplantations were aggregated by calculating the distribution-weighted mean log(PIRCHE-II) values (WML) in previously defined PIRCHE-II ranges* [*^19^*](https://www.zotero.org/google-docs/?0OZkPf)*. The SWML aggregates all respective WML scores per simulation. Lower SWML scores indicate more simulated transplantations being carried out with lower PIRCHE-II scores.*

*The feasibility of our suggested allocation system changes was evaluated by observing PIRCHE-II scores dependent on HLA match grades and vice versa. Also, the distributions of waiting time of patients on the waiting list and transplanted patients, kidney exchange, country balance, recipient age, waiting list size per country, number of transplantations per country, and points given by histocompatibility were taken into account.*

### 3) I suggest also you to include ETKAS, Substituting HLA matching by PIRCHE-II based matching, Full HLA match prioritization, HLA match grade replacements and HLA mismatch probability sub-sections before the presentation of the results which are quite incomprehensible without.

We agree with the reviewer and addressed that point by swapping the sections as suggested in the next issue.

### 4) Anyway, it would be better to have the Material and Methods before the results but I guess this is possible for PLOS computational biology. This section should also separate more clearly what is related to data generation, process simulation (MCMC) and allocation scheme simulation which are the three pillars of you entire "simulation" work.

We agree with the reviewer that placing the Materials and Methods section before the Results section improves the comprehension of the article. We swapped the sections, adjusted the figure and table numbers and highlighted the respective section headings and numbers accordingly.

### 5) The introduction is clear, maybe should you end with what can be expected in terms of feasibility and benefit for ETKAS. It could also starts with the fact that we are in the context of Kidney transplantation.

We thank the reviewer for the suggestion and adjusted the last paragraph or the introduction as follows (lines 172ff):

*“In the present study, we simulated Eurotransplant’s largest kidney allocation program to evaluate the impact of including T-cell epitope matching. To this end, HLA-related factors in ETKAS were converted to PIRCHE-dependent factors. These PIRCHE-dependent factors were optimized in this study to achieve a high epitope matching result, which is expected to improve kidney graft survival of transplanted patients. The feasibility of our approach was concluded by observing a small impact on non-HLA outcome parameters, such as waiting time, waiting list size, and country balance.”*

### 6) Of note, some of bibliographical references in the text goes to strange references according to the statement in the text and the paper it refers to. Could you check this.

We thank the reviewer for carefully checking and apologize for the mistakes. Indeed, some references pointed to the wrong entry of the bibliography due to some final preparations before submission. We checked all references and corrected them accordingly.

### 7) The discussion is also very interesting. I suggest you state as an underlying but very reasonable hypothesis that epitope matching is better than actual serological matching.

As suggested by the reviewer, we added this statement to the discussion (lines 591f):

*“Therefore, allocation based on matching for epitope compatibility rather than matching for serological antigen may be beneficial.”*

### I found pretty smart the way you generated Ab in the recipients (forbidden antigens). But for many immunologists it looks that the number of eplets related to an epitope might be misleading. What matters is the 3D conformation of HLA molecule. To avoid the apparition of de novo DSA after KTx is according to epitope mismatch is the real objective, that needs a prospective evaluation.

We agree with the reviewer, that the immunogenicity and clinical impact of antibody epitopes based on the tertiary structure of the HLA (i.e. eplets) is still controversially discussed. We considered eplets only for the simulation concept of HLA presensitization in a qualitative fashion. The quantity of eplets was neither considered in constructing the virtual cohorts nor in simulation. Consequently, the same *number* of eplets may correspond to a wide immunization (high PRA levels, wide antibody profile) or a narrow immunization (low PRA levels, few HLA antibodies). We clarified this in the Materials and Methods (lines 219f):

*“Given each eplet’s individual frequency, target PRA levels may be reached with low or high eplet counts.”*

Numbers of allo-peptide presented via the indirect pathway (i.e. PIRCHE, more generally T cell epitopes) were previously shown to correspond with increased incidence of dnDSA post KTx. It is well known, the indirect pathway of allo-recognition is a mediator of graft rejection. However, potential immunogenicity of individual peptides, T cell binding affinity and additive effects of B cell and T cell epitopes are still not fully understood. Clearly, the overall objective of epitope matching is a clinical benefit of transplanted recipients realized by lowered dnDSA incidence. We clarified the necessity to observe the beneficial clinical effect prospectively (lines 616f):

*“Similarly, introducing epitope matching techniques to the current allocation system requires thorough monitoring of the expected clinical impact by e.g. monitoring DSA incidence and the respective epitope scores.”*

### In the introduction, you state that "recent studies have shown that predicted donor-HLA epitopes presented on HAL class-II molecules ... are related to HLA antibody formation. I suggest you add the references to theses studies and move this point in the discussion.

We added the references accordingly and included new references to recent articles that were published in the meantime (lines 155ff):

*“Recent studies have shown that predicted donor-HLA derived epitopes presented on HLA class-II molecules, designated as PIRCHE-II, are related to HLA antibody formation* [*^19,22^*](https://www.zotero.org/google-docs/?Ucx4iP)*. Moreover, other retrospective organ transplantation studies using the PIRCHE algorithm have shown that T-cell epitope matching indeed leads to an improved transplant outcome* [*^20,23^*](https://www.zotero.org/google-docs/?gt4V7l)*.”*

Furthermore, we added these references to the first paragraph of the discussion (lines 590f):

*“Epitope matching can significantly improve the outcome of HLA-mismatched kidney transplantation* [*^19,20,22,23,31^*](https://www.zotero.org/google-docs/?fDsPGk)*.”*

### Any future works ? What about reproducibility of in silico experimentations in other countries ?

We thank the reviewer for reminding us to mention the important outlook and a statement on generalizability. Accordingly we added to the discussion (lines 719ff):

*“A collaborative project was initiated with Eurotransplant to apply comparable simulations of ETKAS to their retrospective transplant cohort. This project may be extended to cover also the AM allocation.”*

Applying the simulations to allocation systems in other countries is an option, though being challenging due to the complex nature of these systems. Accordingly we added to the discussion following paragraph (lines 654ff):

*“Focusing on very specific aspects of the highly complex ETKAS, our allocation models are highly tailored to Eurotransplant’s kidney allocation. Nevertheless, basic concepts apply to other transplantation networks in other regions likewise (e.g. virtual populations, patient flow, waiting list management, etc. )and may therefore be reused and adapted accordingly. Also, our simulations may serve as a basis to evaluate more profound changes of the allocation paradigms with respect of utility and equity. Given the variability of organ allocation rules in different countries, adapting simulations or providing a general simulation framework remains however challenging.”*
