# Supplementary material for: Computational Eurotransplant kidney allocation simulations demonstrate the feasibility and benefit of T-cell epitope matching
Source: PLoS Comput Biol. 2021 Jul 27;17(7):e1009248. doi: 10.1371/journal.pcbi.1009248 (PMC8345832; doi:10.1371/journal.pcbi.1009248)
Supplement: Attachment — Submitted filename: Point by point reply rev 2.docx [file pcbi.1009248.s003.docx]

Dear Editors, dear Reviewers,

We appreciate the feedback of the reviewers with respect to our manuscript. Based on the further comments, we improved the manuscript. The corresponding changes of this second revision are highlighted with a blue background, keeping the changes by the first revision highlighted in yellow. Thank you very much for considering our manuscript further.

Sincerely

Matthias Niemann

Point-by-point reply:

Reviewer #1:

Thank you for further explaining your simulation methods and addressing my previous comments.

You said that "We think it is not to be expected that epitope matching is more prone to “racial allocation” than plain serologic matching."

Let me explain what I meant. The population on the waitlist is not necessarily racially distributed similarly to those of a marrow donor program. We know that the risk of kidney disease is not equal across races, and that race is linked with haplotype, blood group, and patient survival among kidney disease patients. While all of these may impact the validity of your simulation, what I was referring to was the idea that changes in the allocation system that are related to haplotype distribution may affect the racial distribution of the recipients. I realize you don't have the ability to describe this with your data, but you should mention it as a possibility that should be investigated, unless there's something I'm not understanding.

We agree with the reviewer, that e.g. disease-susceptibility related to ethnicity (or more specifically its underlying genotypes) may impact our simulations. An option to overcome that would be the usage of real-world data, which is planned in a follow-up project with Eurotransplant as pointed out in the manuscript.

The reviewer also brings up the important point, that HLA-type-related changes to the allocation may impact the composition of recipient genotypes on the waiting list. E.g. by a preference of the allocation algorithm to transplant genotype A, consequently less recipients with genotype A are on the waiting list, with a potential for piling up patients with genotypes B and C. Given the relation between genotypes and ethnicity, this might lead to disadvantages for a population over another.

Though we agree with the reviewer that careful attention should be applied, Eurotransplant’s concept of “Mismatch Probability” is designed to counteract such disadvantages. In our adapted ETKASPIR-E/-F models, 400 points were allocated based on PIRCHE-II matching between patient and donor. However, via the PIRCHE-II Risk Profile, 100 points were allocated based on the probability of the patient receiving a well matched donor offer in the present donor population. Therefore, a disadvantage in match points consequently will lead to an advantage in points for mismatch probability.

To stress the importance of this issue and provide additional clarity, we added another paragraph to the manuscript (lines 701ff):

*“It must be acknowledged that changing matching rules dependent on individuals’ genotypes may cause advantages/disadvantages for certain subpopulations. These advantages and/or disadvantages may lead to an increase in certain subpopulations on the waiting list. To promote more equality for such situations, Eurotransplant implemented the MMP – a correction factor that adds additional points to patients that are less likely to receive a well-matched transplant.”*

Reviewer #2: Thank you for the revised version which is really improved. Some minor changes could be added.

(1) maybe a reference for Gibbs sampler.

Thank you for pointing this out. An appropriate reference was added (line 225):

*“The core of the implemented simulation was a Gibbs sampler* [*[30]*](https://www.zotero.org/google-docs/?0bdFd5) *that considered the virtual population’s characteristics to create a sequence of transplantation events and waiting list snapshots.”*

[*30.	Geman S, Geman D. Stochastic relaxation, gibbs distributions, and the bayesian restoration of images. IEEE Trans Pattern Anal Mach Intell. 1984 Jun;6(6):721–41.*](https://www.zotero.org/google-docs/?NH9Hq3)

(2) I see that DQA is not taken into account. This might be a future work as epitopes for Ab anti DQ are often located on a common zone of alpha and beta chain. DQB matching only might no be ideal or its relevance could be challenged, even at epitaphic level.

We agree with the reviewer and also opt for improved typing methodologies being applied in solid organ transplantation. As most retrospective datasets analysed so far were restricted to HLA-A, -B, -C, -DRB1 and -DQB1, there is little clinical evidence so far, with certain questions not yet fully addressed (e.g. normalizing heterodimer presentation scores with variable alpha chain) to suggest applying an enhanced PIRCHE-II version in allocation practice yet. Additionally, haplotype data that includes HLA-DQA1 is sparse. We stated in the discussion, that the implemented workflow can well be extended to more complex HLA typing data, once there is ample clinical evidence and reference typings are available (lines 743ff):

*“Our simulations considered PIRCHE-II scores with HLA-A, -B, -C, -DRB1 and -DQB1 as peptide sources, with HLA-DRB1 as the only presenting locus. With multiple imputation enabled, providing HLA-A, -B and -DRB1 is sufficient to calculate reasonably accurate PIRCHE-II scores.* [*[51]*](https://www.zotero.org/google-docs/?1GrO4n) *This matches the requirements of the current Eurotransplant allocation rules, potentially reducing the burden of implementing suggested changes. However, there is growing evidence of the clinical relevance of presentation of allopeptides by e.g. HLA-DQA1/-DQB1 heterodimers to CD4+ T cells.* [*[22]*](https://www.zotero.org/google-docs/?kOB7B1) *After further clinical validation, implementation of corresponding typing methods in the pre-transplant diagnostic routine and upon availability of haplotype frequency datasets including HLA-DQA1 and HLA-DP, the presented workflow might be adapted to simulate enhanced PIRCHE-II scores’ impact on allocation.”*

(3) PIRCHE-II RP : RP acronym = ?

Thank you for pointing this out. We added the according explanation of the abbreviation to line 342:

*“Analogous to this system, the PIRCHE-II Risk Profile (PIRCHE-II RP) considers the likelihood of a patient being well-matched from the perspective of the PIRCHE-II score.”*

(4) In the evaluation metrics section, you could just state for the reader that low SWML score means better matching or not. In the discussion: iI suggest you to replace "there may be additional factors that affect the simulation outcomes" with "there may be additional factors that affect the realism of simulation results".

We thank the reviewer for his suggestions and amended accordingly (lines 384f and line 684):

*“Lower SWML scores indicate more simulated transplantations being carried out with lower PIRCHE-II scores (i.e. lower SMWL is better).”*

*“Despite the fact that simulations can reflect real-life situations, there may be additional factors that affect the realism of simulation outcomes.”*

(5) "Transplantable" and "Not Transplantable" could be replaced by "Active" and "Inactive" status.

We understand the reviewer's suggestion to use the more common “active/inactive” nomenclature. We adjusted the terminology but kept the official Eurotransplant nomenclature in parenthesis (lines 695f):

*“Similarly, the status of patients on the waiting list is dynamic between* “active” (transplantable, T) and “inactive” (“not-transplantable”, NT).*”*
